# Coronary plaque tissue characterization in patients with premature coronary artery disease

**DOI:** 10.1007/s10554-020-01794-9

**Published:** 2020-02-20

**Authors:** Jianchang Xie, Jie Qi, Hengyi Mao, Ningfu Wang, Xianhua Ye, Liang Zhou, Guoxin Tong, Jianmin Yang, Hao Pan, Jinyu Huang

**Affiliations:** 1 Department of Cardiology, Affiliated Hangzhou First People’s Hospital, Zhejiang University School of Medicine, 261# Huansha Road, Shangcheng District, Hangzhou, 310006 China; 2 Department of Cardiology, Wenzhou People’s Hospital, Wenzhou, China; 3 Department of Cardiology, Ningbo First Hospital, Ningbo, China

**Keywords:** Premature coronary heart disease, iMap intravascular ultrasound, Cardiac catheterization, Plaque

## Abstract

Premature coronary artery disease (CAD) studies rarely involve coronary plaque characterization. We characterize coronary plaque tissue by radiofrequency intravascular ultrasound (IVUS) in patients with premature CAD. From July 2015 to December 2017, 220 patients from the Department of Cardiology, Affiliated Hangzhou First People’s Hospital, Zhejiang University School of Medicine with first occurrence of angina or myocardial infarction within 3 months were enrolled. Patients with premature CAD (n = 47, males aged < 55 years, and females aged < 65 years) or later CAD (n = 155) were retrospectively compared for cardiovascular risk factors, laboratory examination findings, coronary angiography data, gray-scale IVUS, and iMap-IVUS. The mean age was 53.53 ± 7.24 vs. 70.48 ± 8.74 years (p < 0.001). The groups were similar for traditional coronary risk factors except homocysteine (18.60 ± 5.15 vs. 17.08 ± 4.27 µmol/L, p = 0.043). After matching for baseline characteristics, LDL cholesterol (LDL-C) was higher for premature CAD than later CAD (2.50 ± 0.96 vs. 2.17 ± 0.80 mmol/L, p = 0.019). Before the matching procedure, the premature CAD group had shorter target lesion length [18.50 (12.60–32.00) vs. 27.90 (18.70–37.40) mm, p = 0.002], less plaque volume [175.59 (96.60–240.50) vs. 214.73 (139.74–330.00) mm^3^, p = 0.013] than the later CAD group. After the matching procedure, the premature CAD group appeared to be less plaque burden (72.69 ± 9.99 vs. 74.85 ± 9.80%, p = 0.005), and positive remodeling (1.03 ± 0.12 vs. 0.94 ± 0.18, p = 0.034), and lower high risk feature incidence (p = 0.006) than the later CAD group. At the plaque’s minimum lumen, premature CAD had more fibrotic (p < 0.001), less necrotic (p = 0.001) and less calcified areas (p = 0.012). Coronary plaque tissue was more fibrotic with less necrotic and calcified components in premature than in later CAD, and the range and degree of atherosclerosis were significantly lower.

## Introduction

Coronary artery disease (CAD) is the leading cause of mortality and much is known about the causes and risk factors for the disease [1]. CAD is a progressive disease that takes time to develop [2]. Therefore, age is a significant contributory factor for CAD [3]. When CAD occurs in younger than expected patients it is considered to be premature CAD. However, the definition of premature CAD is difficult because of the variation in risk factors in different populations [4]. Chinese studies use the definition of men with onset age < 55 years, and women with onset age < 65 years [5]. The risk of developing CAD at a younger age appears to occur more often in Asian populations and particularly in South Asians [6], and is related to the established risk factors including thrombotic (smoking, low fruit/vegetable intake, fibrinogen, homocysteine) and atherosclerotic (hypertension, high fat diet, dyslipidemia) factors in combination with a genetic basis [7]. In China, despite understanding risk factors, the incidence of young CHD, in people ≤ 45 years, is increasing [5].

Prior studies have examined the relationship between a family history of premature CAD and coronary artery calcium [8], plaque burden [9] in healthy relatives from families with premature CAD by computed tomography angiography (CTA). Coronary angiography is a well-established diagnostic modality for percutaneous coronary intervention (PCI) guidance, but more recent intravascular imaging techniques enhance the efficacy of lesion evaluation [10]. For example, advanced imaging has shown that the necrotic tissue volume is a potent predictor of periprocedural myocardial infarction after PCI [11]. Importantly, the incidence of an acute coronary event is determined by the extent and severity of the luminal stenosis, especially the characteristics of the plaque [12]. intravascular ultrasound (IVUS) has been developed to access plaque composition and define atherosclerotic lesion phenotype [13]. Classical greyscale IVUS has now been expanded with spectral analysis of back scattered radio frequency (RF) data. Based on pattern recognition of the RF signals, iMap-IVUS (Boston Scientific, Marlborough, MA) can provide quantitative analysis of plaque composition and classify them to four tissue types (fibrotic, lipidic, necrotic, and calcified) in vivo [14, 15].

While most studies on premature CAD focus on genetics and epidemiology, there are few studies related to coronary plaque characterization. In consideration of the genetic basis of premature CAD, whether patients with premature CAD exhibit differences in plaque characteristics is currently not known. Therefore, an investigation into the plaque characteristics of premature CAD and how they compare with typical CAD might reveal important information on the development of this disease.

This study aimed to analyze the morphological, tissue, and phenotypical characteristics of atherosclerotic plaques using iMap-IVUS in patients with premature CAD and compare the differences with patients with CAD who developed the disease later.

## Methods

### Study patients and materials

In this single center study, from July 2015 to December 2017, a total of 220 patients from the Department of Cardiology, Affiliated Hangzhou First People’s Hospital, Zhejiang University School of Medicine with first occurrence of angina or MI in 3 months were enrolled. These were consecutive patients who underwent diagnostic coronary angiography or PCI and were diagnosed with acute coronary syndrome (ACS) or stable CAD.

The inclusion criteria were as follows: (1) First occurrence of angina or myocardial infarction (MI) in 3 months. (2) Diagnosed with ACS or stable CAD by coronary angiography (CAG). The exclusion criteria were as follows: (1) Cholesterol-lowering therapy more than 1 month. (2) CAG showed in-stent restenosis and vision thrombus. (3) Inadequate imaging quality.

The patients were grouped into the premature CAD group and later CAD groups according to their age at diagnosis. Premature CAD was defined as patients aged less than 55 years in men and less than 65 years in women [5]. The older patients were defined as the later CAD group.

### Intravascular ultrasound

The IVUS data were acquired with an iLab System and mechanical 40 MHz IVUS catheter (Both Boston Scientific, Natick, MA, USA). The catheter was advanced beyond the target lesion, and imaging was performed during automatic pullback at a speed of 0.5 mm/s. RF signals were captured on every 4th IVUS frame for tissue compositional data. All recordings were assigned to randomly generated examination ID numbers corresponding to a list and archived to CD-ROM for later offline analysis. Based on the criteria of the American College of Cardiology Clinical Expert Consensus Document on IVUS [16], two experienced analysts who were unaware of the angiographic findings or the baseline clinical and lesion characteristics analyzed the gray-scale IVUS images. QIvus (iMap Basic Viewer 3.0, Medis medical imaging systems bv, Leiden, the Netherlands) was used for analysis.

In ACS group,culprit lesion was identified as target lesion. In stable CAD group, target lesion was defined as the lesion with the most severe plaque burden or needed be intervened firstly in multivessel CAD. In each target lesion, a cross sectional area (CSA) for the lumen and the external elastic membrane (EEM) was traced manually. Plaque burden was calculated as the ratio between plaque CSA (EEM minus lumen CSA) and EEM. Lumen and vessel volumes were calculated as the summation of lumen and EEM area in each measured image respectively. Total atheroma volume (TAV) was calculated as vessel volume − lumen volume. Percent atheroma volume (PAV) was calculated as (TAV/vessel volume) × 100%.

iMap software-classified plaque into four tissue components and produced color images (green for fibrous plaque, yellow for lipidic plaque, pink for necrotic plaque and blue for calcified plaque). A plaque that was unsuitable for analysis was defined as an acoustic shadowing area and volume behind calcification or wire artifact [17].

### Statistical analysis

Categorical variables are presented as percentage frequencies and were analyzed using χ^2^ tests or Fisher exact tests as appropriate. Continuous variables are expressed as mean ± standard deviation (SD) or median (IQR) and were compared using the student’s two-tailed unpaired *t* test or Mann–Whitney test.

We performed a matching procedure to account for differences in baseline characteristics between premature CAD and later CAD. Every patient with premature CAD was matched to the one with later CAD by gender, body mass index (BMI), indication for catheterization (ACS or stable CAD), history of hypertension, diabetes mellitus, statin use, and current smoking.

Data analysis was performed with the Statistical Package for Social Sciences (SPSS) software on Windows version 24.0 (IBM corp. Armonk, NY, USA). All statistical tests were two-tailed and p-values < 0.050 were considered statistically significant.

## Results

### Patients baseline characteristics

From the 220 patients selected for inclusion in the study, 18 patients were excluded for in-stent restenosis, vision thrombus with angiographically, and an inadequate imaging quality. Finally, 202 patients were analyzed.

The baseline clinical characteristics of the total patient population are presented in Table 1. There were 47 patients in the premature CAD group and 155 patients in later CAD group. The mean age was 53.53 ± 7.24 vs. 70.48 ± 8.74 years respectively (p < 0.001). There were no significant differences between the two groups in terms of traditional coronary risk factors except homocysteine (18.60 ± 5.15 vs. 17.08 ± 4.27 µmol/L, p = 0.043). Due to the different diagnostic criteria of premature CAD for different genders, the males in the premature CAD group was significantly lower than in the later CAD group (51.06 vs. 70.97%, p = 0.014). After the matching procedure, baseline clinical and procedural characteristics were similarly distributed between the two groups. The level of low-density lipoprotein (LDL) cholesterol (LDL-C) of the premature CAD group was significantly higher than for the propensity score matched later CAD group (2.50 ± 0.96 vs. 2.17 ± 0.80 mmol/L, p = 0.019). Renal function was significantly better in the premature CAD group when compared with the later CAD group (76.58 ± 15.96 vs. 68.80 ± 14.02 mL/min/1.73 m^2^, p < 0.001) (Table 1). Obviously, eGFR is closely related with age, a comparison between same age groups would be more suitable.

**Table 1:** Baseline clinical characteristics

	Premature CAD (n = 47)	Later CAD (n = 155)	p-value	Propensity score^a^ matched later CAD (n = 47)	p-value
Patient characteristics					
Age, years	53.53 ± 7.24	70.48 ± 8.74	< 0.001	72.72 ± 8.55	< 0.001
Male gender	24 (51.06%)	110 (70.97%)	0.014	18 (38.30%)	0.530
Body mass index, kg/m^2^	23.87 ± 3.13	23.55 ± 3.18	0.545	24.20 ± 3.41	0.348
Current smokers	16 (34.04%)	67 (43.23%)	0.262	13 (27.66%)	0.652
Hypertension	29 (61.70%)	107 (69.03%)	0.348	32 (68.09%)	0.517
Diabetes mellitus	14 (29.79%)	39 (25.16%)	0.528	11 (23.40%)	0.804
Heart failure	8 (17.02%)	45 (29.03%)	0.198	15 (31.91%)	0.190
Premature CAD family history	8 (17.02%)	22 (14.19%)	0.683	3 (6.38%)	0.153
Statin use	10 (21.28%)	52(33.55%)	0.232	14 (29.79%)	0.466
Homocysteine, µmol/L	18.60 ± 5.15	17.08 ± 4.27	0.043	18.13 ± 5.74	0.677
Acute coronary syndrome,	26 (55.32%)	97 (62.58%)	0.372	31(65.96%)	0.826
eGFR-CKD-EPI, mL/min/1.73 m^2^	76.58 ± 15.96	73.16 ± 15.49	0.189	68.80 ± 14.02	< 0.001
HbA1c, %	5.60 (5.30–6.60)	5.70 (5.30–7.00)	0.846	5.80 (5.40–7.00)	0.477
Lipid profile at baseline, mmol/L					
Total cholesterol	4.19 ± 1.18	3.89 ± 1.08	0.107	4.01 ± 0.89	0.424
HDL cholesterol	1.08 ± 0.29	1.06 ± 0.54	0.807	0.99 ± 0.21	0.838
LDL cholesterol	2.50 ± 0.96	2.24 ± 0.88	0.084	2.17 ± 0.80	0.019
Triglyceride	1.83 ± 1.08	1.55 ± 0.84	0.072	1.82 ± 0.96	0.759
C-reactive protein, mg/L	4.00(3.00–6.00)	3.00(3.00–5.00)	0.386	4.00(3.00–5.00)	0.150

Data are expressed as mean ± standard deviation or median (interquartile range) or number (%)

*CAD* coronary artery disease, *eGFR* estimated glomerular filtration rate, *CKD* chronic kidney disease, *EPI* exocrine pancreatic insufficiency, *HbA1c* glycated hemoglobin, *HDL* high-density lipoprotein, *LDL* low-density lipoprotein, *BMI* body mass index, *ACS* acute coronary syndrome

^a^Propensity score was matched by gender, BMI, indication for catheterization (ACS or stable CAD), hypertension, diabetes mellitus, statin use, and current smoking

### Plaque characteristics from coronary angiography

The plaque characteristics as classified by coronary angiography are summarized in Table 2. For the whole patient population, the target lesions occurred most often in the left anterior descending artery. It seems that proportion of complex lesions in later CAD group was higher than that in premature CAD group, but the difference was not significant after the matching procedure (Table 2).

**Table 2:** Plaque characteristics from coronary angiography

	Premature CAD (n = 47)	Later CAD (n = 155)	p-value	Propensity score^a^ matched later CHD (n = 47)	p-value
Target artery			0.870		0.705
LAD	29 (61.70%)	92 (59.35%)		25 (53.19%)	
RCA	10 (21.28%)	36 (23.23%)		12 (25.53%)	
LCX	8 (17.02%)	27 (17.42%)		10 (21.28%)	
Complex lesion B2C	9 (19.1%)	55 (35.5%)	0.035	11 (23.40%)	0.614
Number of diseased vessels	1.00 (1.00–2.00)	1.00 (1.00–2.00)	0.762	2.00 (1.00–3.00)	0.563

Data are expressed as median (interquartile range) or number (%)

*CAD* coronary artery disease, *LAD* left anterior descending, *RCA* right coronary artery, *LCX* left circumflex

^a^Propensity score was matched by gender, BMI, indication for catheterization (ACS or stable CAD), hypertension, diabetes mellitus, statin use, and current smoking

### Plaque characteristics from iMap-IVUS

**Fig. 1:**
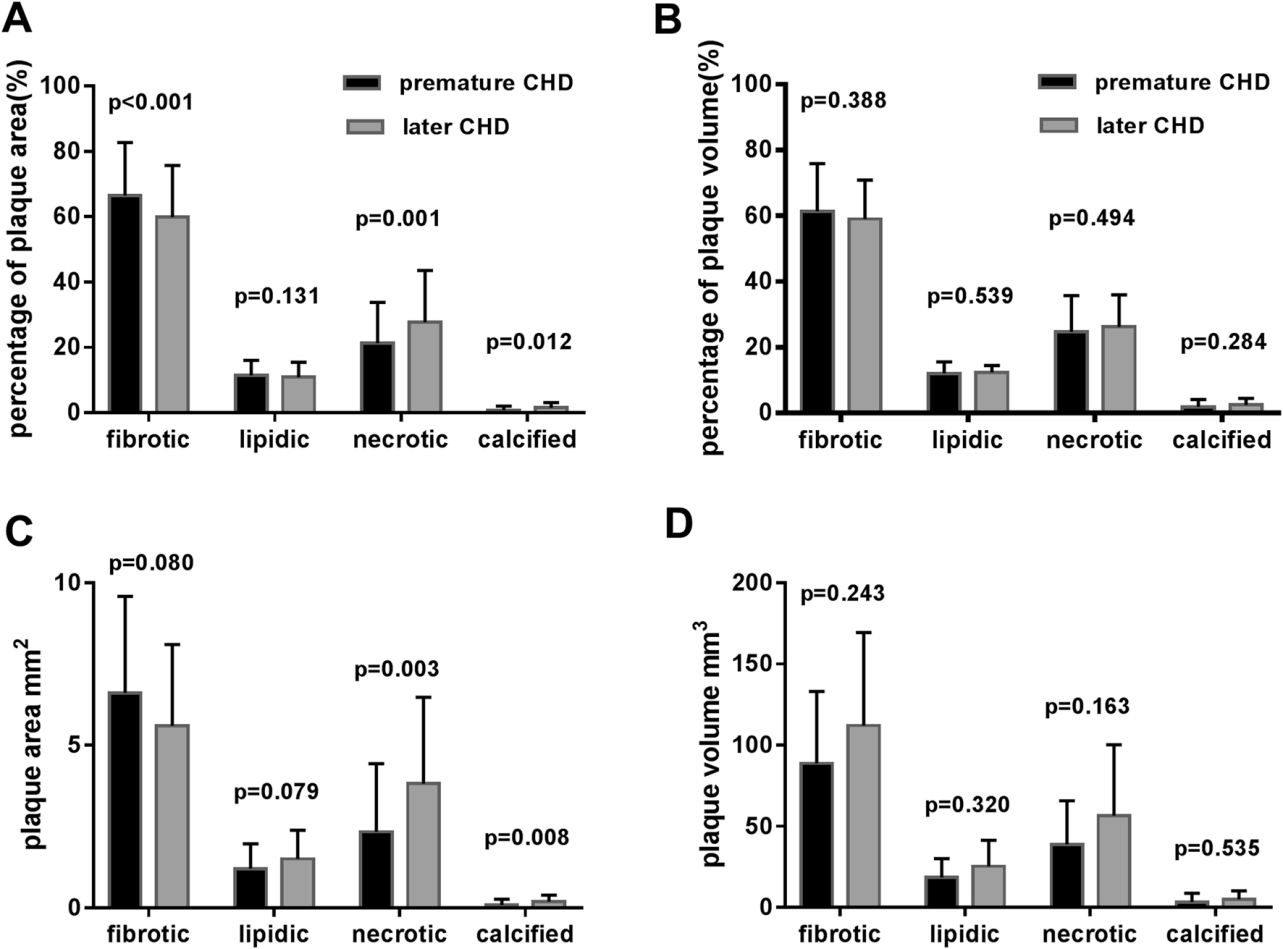
At the minimum lumen cross sectional area (CSA) site in each plaque, the percent fibrotic was higher, percent necrotic and calcified was lower in premature CAD group than later CAD (**a**). Between the two groups, there were significant differences in necrotic and calcified areas (**b**). In the whole plaque, the difference of plaque components between the two groups was not significant (**c**). The mean plaque volume in the later CAD group was greater than that in the premature CAD group, and every component was greater too (**d**)

The iMap-IVUS data are listed in Table 3. Before the matching procedure, the target lesion length in the premature CAD group was shorter than that in the later CAD group [18.50 (12.60–32.00) vs. 27.90 (18.70–37.40) mm, p = 0.002], besides, the plaque volume was much less in the premature CAD group [175.59 (96.60–240.50)] vs. 214.73 (139.74–330.00)mm^3^, p = 0.013], although these differences were not significant after the matching procedure. Furthermore, in the premature CAD group, the plaque burden was lower compared to the later CAD group (72.69 ± 9.99 vs. 74.85 ± 9.80%, p = 0.005) with less negative remodeling (1.03 ± 0.12 vs. 0.94 ± 0.18, p = 0.034). Overall, the proportion of each of these 4 plaque components was significantly different between the premature and later CAD groups. At the minimum lumen CSA site in each plaque, the premature CAD appears to have more fibrotic area (66.47 ± 16.26 vs. 53.06 ± 19.45, p < 0.001), less necrotic area (21.28 ± 12.51 vs. 27.70 ± 15.84%, p = 0.032) and significantly less calcified area [0.04 (0.02–0.12) vs. 1.56 (0.42–3.49), p = 0.012] than in the later CAD group. However, the difference in plaque components for the whole plaque was not significant (Fig. 1). Besides, the incidence of high risk features: plaque burden of ≥ 70%, and iMap IVUS derived thin-cap fibroatheroma (TCFA), were significantly lower in premature CAD group (72.34 vs. 89.36%, p = 0.036, 59.57 vs. 85.11%, p = 0.006 respectively) (Table 3).

**Table 3:** iMap-IVUS segment and lesion characteristics in the target artery

	Premature CAD (n = 47)	Later CAD (n = 155)	p-value	Propensity score^a^ matched Later CHD (n = 47)	p-value
Segment length, mm	18.50 (12.60–32.00)	27.90 (18.70–37.40)	0.002	24.60 (15.50–36.00)	0.072
iMap IVUS analysis (2D)					
Lumen CSA^b^, mm^2^	3.63 ± 1.49	3.36 ± 1.99	0.404	3.12 ± 1.89	0.154
EEM CSA^b^, mm^2^	12.78 (10.37–17.20)	13.96 (11.13–16.41)	0.467	13.10 (11.01–15.81)	0.719
Plaque CSA^b^, mm^2^	10.23 ± 4.30	10.89 ± 4.08	0.342	11.12 ± 4.25	0.319
Plaque burden, %	72.69 ± 9.99	75.97 ± 10.46	0.059	74.85 ± 9.80	0.005
Remodeling index	1.03 ± 0.12	0.95 ± 0.24	0.029	0.94 ± 0.18	0.034
Fibrotic area, %	66.47 ± 16.26	51.68 ± 19.97	< 0.001	53.06 ± 19.45	< 0.001
Lipidic area, %	11.49 ± 4.58	12.54 ± 4.76	0.185	12.98 ± 4.89	0.131
Necrotic area, %	18.32 (10.05–30.93)	30.25 (20.24–49.33)	< 0.001	30.84 (20.04–47.33)	0.001
Calcified area, %	0.43 (0.19–1.22)	1.26 (0.38–3.14)	0.002	1.56 (0.42–3.49)	0.012
Fibrotic area, mm^2^	6.61 ± 2.98	5.33 ± 2.39	0.003	5.60 ± 2.50	0.080
Lipidic area, mm^2^	1.20 ± 0.77	1.42 ± 0.88	0.119	1.50 ± 0.89	0.079
Necrotic area, mm^2^	1.79 (0.89–4.14)	4.00 (1.46–7.02)	0.002	3.45 (1.54–5.52)	0.003
Calcified area, mm^2^	0.04 (0.02–0.12)	0.12 (0.04–0.29)	< 0.001	0.10 (0.05–0.25)	0.008
iMap-IVUS analysis (3D)					
Lumen volume, mm^3^	87.75 (52.62–156.60)	140.07 (79.17–225.96)	0.005	143.92 (81.92–209.08)	0.036
Vessel volume, mm^3^	238.85 (153.04–389.04)	376.92 (234.80-564.52)	0.004	356.94 (178.36–504.90)	0.080
Plaque volume, mm^3^	175.59 (96.60–240.50)	214.73 (139.74–330.00)	0.013	204.21 (106.70–311.13 )	0.182
PAV, %	62.72 ± 13.11	60.28 ± 11.64	0.222	61.12 ± 11.59	0.569
Fibrotic volume, %	61.37 ± 14.50	57.90 ± 13.76	0.136	59.00 ± 11.85	0.388
Lipidic volume, %	12.00 ± 3.54	12.65 ± 2.96	0.210	12.37 ± 2.09	0.539
Necrotic volume, %	24.74 ± 10.98	27.32 ± 11.34	0.173	26.21 ± 9.74	0.494
Calcified volume, %	2.76 (1.00–4.14)	3.23 (1.26–6.21)	0.165	2.30 (1.14–4.73)	0.284
Fibrotic volume, mm^3^	95.89 (52.50–163.43)	122.57 (74.53–188.65)	0.059	125.31 (65.08–163.55)	0.243
Lipidic volume, mm^3^	16.89 (11.97–31.20)	24.32 (15.02–42.00)	0.018	23.14 (12.80–34.98)	0.320
Necrotic volume, mm^3^	29.64 (21.21–69.60)	51.87 (28.73–103.12)	0.004	48.52 (25.68–71.89)	0.163
Calcified volume, mm^3^	4.00 (1.78–7.20)	5.60 (2.62–13.27)	0.031	4.02 (2.06–10.04)	0.535
High risk lesion characteristics					
Plaque burden ≥ 70%	34 (72.34%)	125 (80.65%)	0.223	42 (89.36%)	0.036
MLA ≤ 4.0 mm^2^	33 (70.21%)	117 (75.48%)	0.469	40 (85.11%)	0.083
TCFA	28 (59.57%)	122(78.71%)	0.009	40 (85.11%)	0.006

Data are expressed as mean ± standard deviation or median (interquartile range) or number (%)

*CAD* coronary artery disease, *IVUS* intravascular ultrasound, *BMI* body mass index, *ACS* acute coronary syndrome, *MLA* minimal lumen area, *TCFA* thin-cap fibroatheroma, *PAV* percent atheroma volume, *EEM* external elastic membrane, *CSA* cross sectional area

^a^Propensity score was matched by gender, BMI, indication for catheterization (ACS or stable CAD), hypertension, diabetes mellitus, statin use, and current smoking

^b^Measuring at the minimum lumen CSA site in each plaque would been properly for comparion

## Discussion

**Fig. 2:**
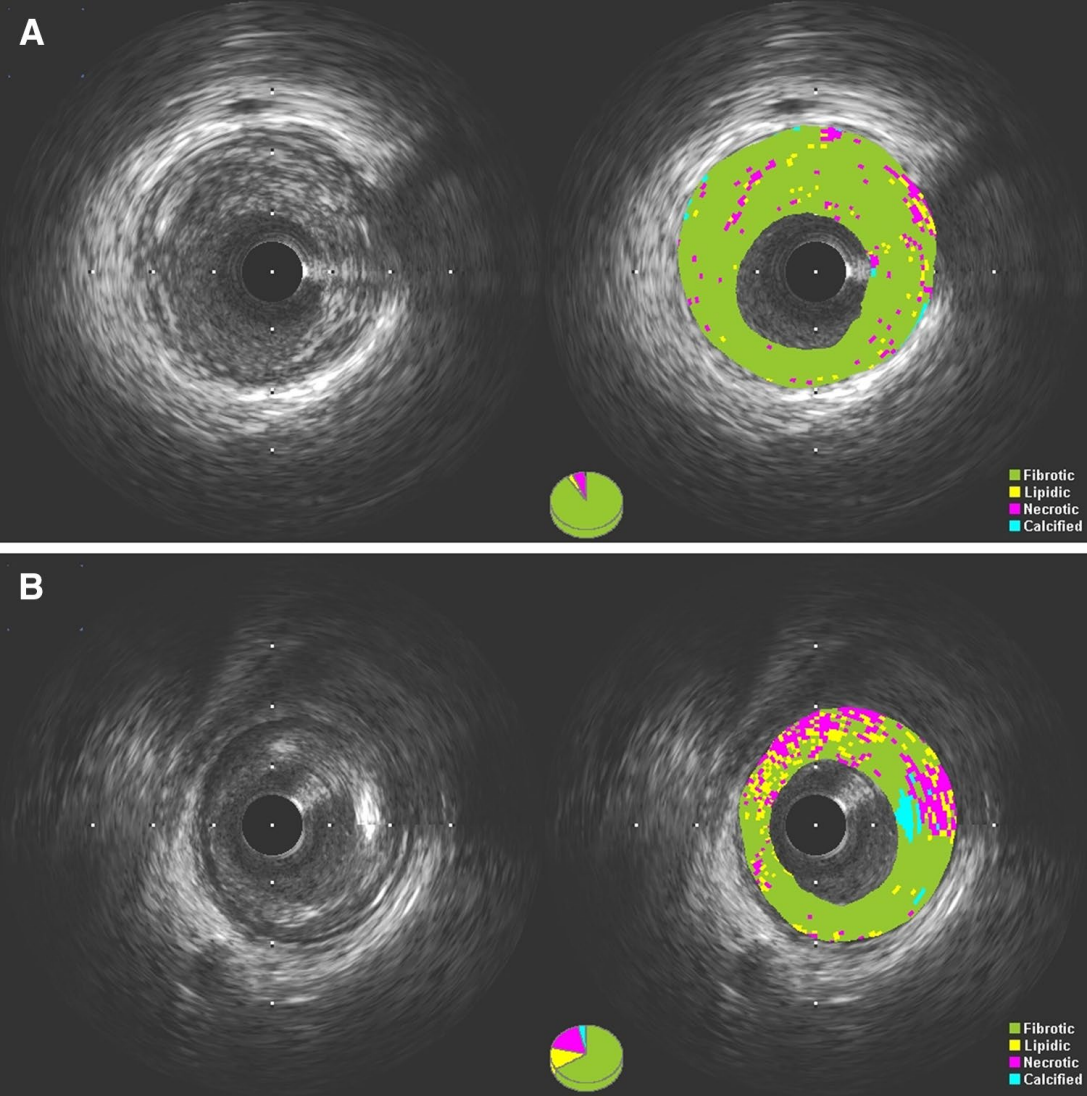
Example of premature CAD plaque characterization at CSA using gray-scale IVUS and iMap-IVUS and compared to the later CAD. **a** A-55-year-old female plaque was composed of fibrotic area (90.89%), lipidic area (2.81%), necrotic area (5.68%) and calcified area (0.62%). **b** A-70-yeas-old male plaque was composed of fibrotic area (66.26%), lipidic area (12.64%), necrotic area (17.72%) and calcified area (3.38%)

The aim of this study was to characterize coronary plaque tissue by iMap-IVUS in patients with premature CAD and compare the results with older patients with CAD. The main findings of the study were as follows: in the premature CAD group compared with the later CAD group, (1) more fibrotic and less necrotic and calcified components were observed. (2) High-risk plaques in terms of plaque burden ≥ 70% and TCFA were observed less in the target lesion. (3) The range and degree of atherosclerosis appears to be lower (number of diseased vessels, lesion length, plaque volume) (Fig. 2).

Previous studies that focused on gender differences showed similar results by Virtual Histology and grey-scale IVUS [18–21]. However, these studies did not match for baseline factors in different aged groups, and these factors may bias the results. Therefore, we focused on plaque characteristics in premature CAD compared with later CAD using propensity score matching for gender, BMI and a few other cardiovascular risk factors. We also controlled the time of onset to within three months to reduce the interference from different disease duration as much as possible. Contrary to these results, a large study found that younger patients had more unstable plaque morphology [21], the possible reasons for the different result are that we excluded patients with visible thrombus and TCFA could not predict prognosis precisely within these limits [12].

Previous grey-scale IVUS studies have demonstrated that elderly patients had more calcified plaques, more negative remodeling and diffuse atherosclerosis [21]. With age vessels become dilated, tortuous, calcified, and dysfunctional, according to classical pathologic and animal model experiments [22, 23]. Young female patients with CAD are rarer than males, and the degree of atherosclerosis was lower in female premature CAD patients. With increasing age, the difference between genders becomes less [18–20]. On the other hand, the plaques of young female patients showed more cellular fibrous tissue and lipid-rich foam cells, which reveals the reversibility of CAD in younger patients [24].

Traditional cardiovascular risk factors are closely associated with premature CAD. Patients with premature myocardial infarction appear to have a higher prevalence of smoking, family history of premature CAD and male gender [25]. Controversially, the degree of hyperlipidemia, hypertension and diabetes does not appear to be as robust as the risk factors already discussed [25]. In this study, there was no significant difference in traditional risk factors between the groups except homocysteine, but after propensity score matching only LDL-C was significantly higher in the premature CAD group. Dyslipidemia plays an important role in development of atherosclerosis, which has been explored a lot in the field of genetics. Compared with the general population, hyperlipidemia is related to autosomal genetic mutations which are characterized by severe elevations in LDL-C which could increase a concomitant 10–20 fold risk of premature CAD [26]. Among these genetic factors, LDL-receptor mutations are the most common genetic defect in all individuals with premature CAD [27]. In the future, proprotein convertase subtilisin-kexin type 9 (PCSK9) inhibitors are expected to ameliorate hyperlipidemia due to genetic causes [28]. For now, statins are effective and economical lipid-lowering therapies. Importantly, these studies suggest that patients with premature CAD could have more benefit from statin therapy than elderly patients [29].

In the general population, serum homocysteine level is associated with age and renal function [30]. However, in this study, premature CAD patients with better renal function and younger age showed a higher level of homocysteine. It should be considered that heredity and metabolize factors are involved in premature CAD [31]. Compared with non-CAD individuals, the level of homocysteine is remarkably higher in patients with CAD especially younger patients, and this has been regarded as an independent risk factor for arteriosclerosis in southern China crowd [32]. Hyperhomocyteinemia is involved in arteriosclerosis through several mechanisms, such as endothelial dysfunction, permeability of cholesterol and inflammatory cells, vascular inflammation, lipoprotein oxidation, smooth muscle proliferation, platelet activation, and abnormalities in the clotting cascade [31, 33, 34]. However, there is much controversy still exists regarding homocysteine as a cardiovascular risk factor. Common genetic variants that influence homocysteine level are not related with risk of CAD in white populations [35]. In fact, most clinical trials focus on supplementation of folic acid with or without vitamin B to ameliorate hyperhomocysteinemia do not reduce the relative risk for CAD [36].

Although this study did not focus on smoking cessation, it seems to be the most important risk factor modification for premature CAD. Current smokers appear to have more lipid-rich plaques than those who had never smoked, or quit more than 1 year ago [37, 38]. After MI, continued smokers with a relative risk of 1.51 (95% CI 1.10–2.07) recurrent coronary events compared with non-smokers, while the risk declined to equal that of nonsmokers 3 years after cessation [39].

This study has some limitations. This was a retrospective study performed at a single center with a relatively small sample size, so selection bias cannot be excluded. In order to avoid the misrecognition of iMap-IVUS to thrombus, some patients with STEMI were excluded. Acoustic shadowing behind calcification or wire artefact makes the analysis incomplete, and these “unknown areas” can often be mistaken for necrotic areas. Different from stable CAD, patients with ACS appears to have less fibrotic, more necrotic and lipidic component [40]. After matching procedure, a higher proportion of patients with ACS in later CAD may affect results. In small sample sizes, propensity match analyses could not exclude some potential cofounders. More samples are required for subgroup analysis in ACS group in future.

## Conclusions

Coronary plaque tissue in premature CAD appears to be more fibrotic with less necrotic and calcified components compared with later CAD, the range and degree of atherosclerosis were significantly lower than in later CAD.
